# Self-Efficacy and the Sense of Coherence: Narrative Review and a Conceptual Synthesis

**DOI:** 10.1100/tsw.2009.107

**Published:** 2009-09-14

**Authors:** Paul Posadzki, Nel Glass

**Affiliations:** ^1^ School of Medicine, Health Policy, and Practice, University of East Anglia, Norwich NR4 7TJ, UK; ^2^ ACU National/St. Vincent's and Mercy Private Hospital, Easthill House, 71 Victoria Parade, Fitzroy, VIC 3065, Australia

**Keywords:** sense of coherence, self-efficacy, narrative review, concept development

## Abstract

In this study, the authors develop an exploratory synthesis of two major health concepts: Antonovsky's sense of coherence and Bandura's beliefs in one's own efficacy. Reinterpretation of each study in the light of the other can lead to greater conceptual development and expand existing knowledge. The mutual themes are presented with an explanation of their contribution to broader conceptual discussions. The existence of some similarities between the two concepts is suggested. Researchers can obtain valuable and additional arguments through cross-fertilization of ideas across presented studies united by shared assumptions. Further research is recommended among various age groups and social backgrounds in order to verify the possible benefits of such theoretical development. Theoretical and practical implications of such a synthesis are presented.

## INTRODUCTION

Antonovsky's concept of the sense of coherence (SOC)[1] is a valuable scientific construct. Similarly, Bandura's theory of an individual's self-efficacy (SE)[2] can be regarded as a key concept applicable to health. Both are widely accepted due to their methodological importance and can be used in order to detect, predict, and change (improve) health status. Due to their relevance and appropriateness in explaining and describing individuals' states of health, both concepts have been given broad attention in the past 30 years.

The review question asks whether generalized resistance resources (GRR) can be developed through SE or whether belief in one's own efficacy may be strengthened by high SOC. The aim of this study is to address this question via description and conceptual synthesis of SE and SOC. Such a compilation may refresh both approaches to human health and bring new insights into the well-being problematic. Patients, therapists, and teachers may also benefit from this fusion.

Evaluation of the SOC construct in the light of SE may be beneficial from the overall well-being perspective in the sense that the addition of SE to SOC may enhance individuals' health resources. Conversely, individuals may develop beliefs in their own capabilities due to increased SOC with and within the Universe. This article reviews similarities and differences between and across diverse studies concerning

SOC and SE in a narrative manner, and suggests some possible benefits of this fusion. The aim of the paper is to generate a conceptual synthesis of SOC and SE. The main areas of convergence are suggested.

## SELF-EFFICACY THEORY

The concept of one's own efficacy that enables an individual to symbolize, learn from others, or plan alternative strategies is based on Bandura's Social Cognitive Theory[2] and cooperates with goal-aiming notion and one's expectations. It is suggested that this concept regulates an individual's motivation, behavior, and mood[3].

SE refers to people's beliefs about their capability to exercise control over the events affecting their lives. SE can also be thought of as the ability to organize and execute the courses of action required to manage prospective situations[3,4,5]. SE beliefs are concerned with judgment about what one can do with whatever skills one possesses[6] and has a complex effect on task performance. Results indicate that positive task experiences elicit a linear increase in SE[7].

Bandura[2] created a concept of self-reflection. This capability is thought to be an insight or self-awareness that is essential for producing enduring behavioral change([2], p.4). This reflection is thought to be a unique human capability that enables individuals to control their thoughts, feelings, and actions, and therefore might be particularly useful in various, e.g., stressful, life events.

It is also worth disseminating that education is an effective variable in improving an individual's SE[8]. Leganger and Kraf[9] state that the higher a person's educational status, the higher his/her SE. Schwarzer and Renner[10] present interesting findings concerning fluctuations of SE during the life span, which suggest that it may grow with age. In terms of gender difference, some authors claim that men report higher levels of SE than women[11]. Other authors suggest that SE differs with racial background[12]. Some researchers suggest that higher SE is strongly associated with higher income[13].

## SELF-EFFICACY AND HEALTH

Giles et al.[14] argue that SE is a key construct in the area of health psychology. Numerous studies verify this statement. A few of these are presented below.

### Self-Efficacy and Health Behaviors

Bandura[3] states that the Theory of Self-Efficacy is a real determinant of an individual's health behaviors and should be mentioned due to its importance. SE may be used as a predictor of health behaviors, such as exercise performance[15,16], cardiorespiratory fitness, weight loss[17], alcohol consumption[18], and oral health[19]. These results support the conceptual and empirical superiority of SE as a predictor of health behaviors and intentions[20].

Some research suggests that SE is important in the initiation and maintenance of a variety of physical activity programs[21,22,23], and a promising and positive indicator presaging sustainability and change in health behaviors[24]. It can be regarded as a closed circle because, conversely, greater physical activity may improve an individual's SE[21].

### Self-Efficacy and Mental Health

Some studies indicate that a belief in SE enables individuals to cope with stress[25]. Others suggest that it is a powerful and modifiable determinant of depressive symptoms that can lead to mental well-being[26]. Sacco et al.[27] support this effect since they found that the effects of poor adherence to antidepressive drugs are mediated by lower SE perceptions. Conversely, greater SE is associated with higher perceptions of health[28].

### Social Benefits of Self-Efficacy

Perkins et al.[29] state that SE can significantly predict social activities. Bandura et al.[5] appear to support this statement, as they have investigated the role of SE as a moderator of affective reactions of psychosocial functioning. Their research shows that SE significantly influences prosocial behaviors via empathy. Furthermore, higher perceived SE scores were associated with increased communication and partnership[30].

Self-presentation of one's own SE via positive health practices can strengthen self-esteem and self-appraisal, and therefore influence health behaviors[31].

### Self-Efficacy in Health Promotion and Disease Prevention

Mosher et al.[32] presented findings that support the use of strategies to increase SE in health promotion interventions for cancer survivors. In terms of sexually transmitted diseases, SE might be effective in order to promote consistent condom use[33]. SE beliefs may facilitate disease prevention and therefore can be regarded as a positive medicine[34].

### Predictive and Prognostic Value of Self-Efficacy

SE is correlated with health expectations and can be considered as a valid predictor of an individual's health practices[10]. Additionally, it has been suggested that SE can be seen as a useful, prognostic tool in the context of performing doctors' prescriptions[35].

## THE CONCEPT OF SENSE OF COHERENCE AS A PART OF GENERALIZED RESISTANCE RESOURCES

Based on the Second Thermodynamics Law (all living organisms can be characterized by the drive to entropy), Antonovsky[1] created the notion of Salutogenesis. This model of health assumes that peoples' health can be situated at some place on the continuous "ease-disease" scale, and is thought to be in opposition to pathogenesis: health as a primordial state.

Aaron Antonovsky created the concept of GRR[1]. GRR are defined as each person's, environment's, or group's characteristics that facilitate effective coping with stress (p. 100). GRR include physical, biochemical (resistance), material, cognitive (knowledge, intellect, personality), and emotional factors associated with values, attitudes, and interpersonal relationships, as well as macrosociocultural qualities of individuals that enable them to avoid or conquer various kinds of stressors efficiently[36]. An individual's level of GRR determines how he/she perceives the world that is being expressed via SOC. According to Antonovsky, SOC is created by a few attitudes that form a kind of a "behavioral immunology" that leads to health[37]. SOC as part of GRR can be defined as a global orientation that creates dynamic and persistent beliefs, and that stimuli from the outside and inside worlds have a logical structure that can be predicted and explained. Individuals' available resources enable them to challenge these stimuli. Challenges are worth engaging and overcoming them may strengthen one's health. Antonovsky claims that the senses of comprehensibility, of manageability, and of meaningfulness are core components of SOC[1]. Comprehensibility (C) is regarded as the belief that incoming stimuli are coherent and make cognitive sense to the individual. Additionally, comprehensibility means that in relation to the future, these incentives will have logic and will be predictable. An individual may perceive manageability (Ma) as resources that he/she is able to use in order to challenge unexpected demands[36]. According to Antonovsky, meaningfulness (Me) is the crucial, motivation-emotional element of SOC. Persons with a high level of this SOC component feel that life makes emotional sense; it is worth engaging and investing energy, devotion, and commitment to. The challenges that sometimes occur are worth confronting and are therefore desired (p. 18). It should be added that Klepp et al.[38] measured within SOC model correlations and proved that Ma, C, and Me were found to be very highly and, in the case of sense of manageability and sense of comprehensibility, even perfectly correlated.

## THE SENSE OF COHERENCE AND HEALTH

Antonovsky's research results indicate a significant correlation between a high level of SOC and overall health[37]. It has been suggested that the SOC is a key construct in the area of health psychology[36]. Recently researchers throughout the world have supported this declaration. Several research results are presented below.

### Sense of Coherence and Health Behaviors

SOC is thought to be an individual's characteristics related to a positive life orientation leading to effective coping strategies[39]. Antonovsky's research shows statistically significant correlations between strong SOC and health behaviors[40]. The results of other studies provide similar conclusions[41,42,43,44,45,46,47,48,49,50,51,52,53].

### Sense of Coherence and Mental Health

Shiber et al.[54] have shown that perception of SOC can ease psychological disorders and reduce the risk of serious disability. Nevertheless, some inconsistencies exist in the literature concerning SOC and its relationship to anxiety and depressive symptoms[55]. These were confirmed when the SOC and coping responses did not mediate the relationship between perceived stress and post-traumatic stress[56,57]. However, other findings show that SOC offers most protective benefits to individuals exposed to symptoms of post-traumatic stress disorder[58]. It has also been shown that a strong SOC alleviates the development of distress[59,60]. Siglen et al.[61] state that SOC is significantly associated with depression; i.e., the stronger the SOC, the lower the levels of depression[62,63,64,65,66] and anxiety[65,67]. It has also been shown that the higher the SOC, the better the mental health of the respondent[68,69]. In addition, a highly significant correlation between psychological health and SOC was found[70,71]. Some findings also suggest that improving SOC in people with mental health problems might provide important opportunities for improving their satisfaction with life [72].

### Social Dimensions of Sense of Coherence

Analyses by Marsh et al.[73] reveal that social support is a strong predictor of SOC. Similarly, social support has been found to be an important cornerstone in the restoration of a person's SOC[74]. The results also suggest that SOC is a potential marker of an individual's social adaptive capacities[75]. Conversely, maladaptive and antisocial behaviors correlate with poor SOC[76].

### Sense of Coherence in Health Promotion and Disease Prevention

It has been suggested that SOC may support a design of future health promotion programs[57,68,77]. Moreover, Simonsson et al.[78] suggested the preventive value of SOC as a resource that strengthens resilience and develops a positive state of health.

### Predictive and Prognostic Value of Sense of Coherence

Longitudinal studies have indicated a predictive value of SOC in terms of health and good quality of life (QOL)[68,79,80,81]. It has also been suggested that health-related QOL[82] and mortality[75] can be predicted using SOC. In addition, individuals with increased QOL have reported higher SOC scores[83]. Bergstein et al.[66] suggested a possible use of SOC as a prognostic tool.

## A CONCEPTUAL SYNTHESIS

The main areas of convergence, theoretical and practical implications, and unaddressed questions are presented below.

### The Main Areas of Convergence

From the health sciences perspective, it can be considered that both theories share similar underlying principles, i.e., their conceptual framework looks similar. C, Me, and Ma may have their equivalents in Bandura's beliefs regarding sense of control over thoughts, feelings, and actions, respectively. Bandura's perception of one's own SE is thought to be a self-regulatory system that influences an individual's thoughts, feelings, and actions. Analogically, Antonovsky's sense of comprehensibility may be equivalent to the cognitive aspect of self-reflection; Antonovsky's sense of manageability may match the behavioral component of Bandura's concept; and finally, Antonovsky's sense of meaningfulness may well correspond to the emotional component of SE. To be more precise, the person's cognitive structures that they can reflect on efficiently are responsible for their vision of the world as coherent, sensible, and rational. Certainly, an individual will carry out appropriate actions and behaviors in order to manage environmental demands. Furthermore, he/she will be able to make sense of stressful life events and this can make a particular individual more "emotionally resistant".

The most explicit common feature of both approaches is that people who have strong beliefs in their capabilities approach difficult tasks as challenges to be mastered and worth confronting, and invest energy in them rather than see them as threats to be avoided[1,3].

1. Both concepts have therapeutic, diagnostic, and predictive value, and both can be used for health promotion and disease prevention.
2. Both can be applied to various dimensions of human health, i.e., psychological, physical, and social.
3. Both SE and SOC can enhance and develop an individual's health, well-being, and QOL.
4. As SOC and SE naturally fluctuate over the human life span, the ongoing development and strengthening of both increases health resources and promotes longevity.
5. Both can be "used" as health mediators and moderators.
6. Both SOC and SE may be developed due to such conceptual synthesis.

## Theoretical Implications of Such a Combination

Bandura ([2], pp. 4–5) writes that the value of a theory is ultimately judged by the power of the procedures it generates to effect psychological change. Moreover, effective theories must demonstrate predictive power.

Psychological changes might be achieved in the sense that SE beliefs, when added into SOC, may strengthen an individual's resources and coping strategies simultaneously. On the other hand, SOC, when incorporated into the SE concept, might develop beliefs in one's own efficacy. Moreover, people with a high SOC might be regarded as more likely to engage in self-reflection concerning their own health. Conversely, individuals with a high level of self-reflection may be more likely to explore their own relationship with the external world.

A combination of SOC and SE may have predictive power in the sense that efficacy beliefs might successfully enhance comprehension of an individual's current and future situation, and his/her ability to give meaning to various impulses bombarding the mind (emotional, cognitive) and to manage them. Furthermore, in terms of SOC's internal consistency, it may be assumed that a higher sense of comprehensibility may interact with and enhance cognitive and self-regulatory processes. The greater his/her sense of manageability or meaningfulness, the more likely an individual is to engage in courses of action required to manage prospective situations. Thoughts can interfere with comprehensibility, feelings with meaningfulness, and actions with manageability. But also within the combined model, interrelations may be observed between thoughts, feelings, and Me, Ma, and C. Manageability may influence the sense of control over thoughts and feelings. Belief in meaningfulness can increase the sense of control over emotions. Analogically, such circumstances may be replicated with regard to manageability and comprehensibility. The conclusion might be as follows: belief in one's own control over thoughts, feelings, and actions may improve Ma, Me, and C.

Certainly, a high level of SE can give an individual the ability to classify the structure of the impulses approaching one's life more effectively. Moreover, increased SE can be used as a strategy for coping with stress. Conversely, categorized impulses and a strong SOC may, in turn, enhance perceived SE. Self-reflection regarding one's own emotional resistance may be useful when some behavioral interventions are required.

## Practical Implications and Guidelines

Policy makers who create a wider health strategy might use a health model based on combined concepts of SE and SOC[84]. For instance, both could be applicable to learning processes in institutions such as schools and academies. This would enable young people to acquire knowledge and skills useful for coping with stress, strengthen their health resources, and enhance their well-being. Both SOC and SE could be part of the explicit curriculum taught continuously at the early stages of ontogenesis, and would give them tools for managing challenging life events.

SE and SOC as a consistent collective may be used in clinical practice, solely or in combination with other therapeutic modalities such Cognitive Behavioral Therapy, Gestalt Therapy, or humanistic approaches, such as patient-centered therapy for patients' best benefits. Moreover, rehabilitation specialists and their cardiology, orthopedic, neurology, pulmonology, and pediatric patients could also benefit from such an incorporation. Exercising control over thoughts, feelings, and actions may be more feasible when one believes in the world's coherence and the logic of one's own existence.

The main practical implications of the combination of both approaches can also be analyzed from a coping strategy perspective. According to Antonovsky, if SOC as behavioral immunology can be mediated, SE is thought to be a key mediator in terms of planning the resources to be used. This would develop the individual's GRR and improve his/her self-confidence and general state of health. SE can be regarded as a moderator of GRR that facilitates an individual's satisfaction with life. Surely, engagement in the process of giving a meaning into an individual's life events should include emotional SE. This would allow development of the individual's emotional resistance or flexibility, and consequently promote social interaction via prosocial behavior, such as altruism and/or empathy.

From the health promotion perspective, SE added to SOC can be used in the process of encouraging people to prevent disease, strengthening their health potential or reservoir, and for persistent self-development[84].

### Questions that Remain Unanswered

1. Does the nature of beliefs need to explored in greater depth since both models are based on them?
2. Would it be scientifically interesting to investigate mutual correlations between sense of manageability and the behavioral component of SE, or sense of comprehensibility and the cognitive "part" of SE?
3. Can pressure toward entropy be minimized to some extent through motivation towards self-organization (reversed entropy) and conscious control over one's own thoughts, feelings, and emotions?
4. Can an individual's health be shifted nearer to the "ease" end of the ease/disease continuum?
5. Can high SE strengthen physical, emotional, cognitive, spiritual, and material resources?
6. How can these two related concepts be used in conjunction with one another?
7. What is the synergistic effect of, for example, high SE and high SOC over and above their main effects? And who tends to experience more under what conditions?

## CRITICAL REFLECTIONS

It appears that Antonovsky's construct does not have strong predictive power in terms of health behaviors. There is also a lack of motivational variables in Antonovsky's model. On the other hand, Bandura's concept of SE does not include a "philosophical component" regarding human existence[2] and SOC, therefore, could be incorporated into SE in order to increase its completeness.

Future research to investigate the correlations between SOC and SE among various age groups with different educational status and social backgrounds should be performed to measure the validity, reliability, and specificity of the new construct.

Of course, such conceptual synthesis may be questioned, but it seems that a unified theory in the area of health psychology/sciences would be a useful adjunct to current and future research.
